# Online self-compassion training to improve the wellbeing of youth with chronic medical conditions: protocol for a randomised control trial

**DOI:** 10.1186/s12889-020-8226-7

**Published:** 2020-01-28

**Authors:** Amy Finlay-Jones, Mark Boyes, Yael Perry, Fuschia Sirois, Rachael Lee, Clare Rees

**Affiliations:** 1 Telethon Kids Institute, PO Box 855, West Perth, Western Australia 6872 Australia; 2 School of Psychology, Curtin University, GPO Box U1987, Perth, Western Australia 6845 Australia; 3 University of Western Australia, 35 Stirling Hwy, Crawley, WA 6009 Australia; 4 Department of Psychology, University of Sheffield, Cathedral Court, 1 Vicar Lane, Sheffield, S1 2LT UK

**Keywords:** Chronic illness, Chronic medical conditions, Youth, Self-compassion, Online, Digital health

## Abstract

**Background:**

Chronic medical conditions (CMCs) affect up to 35% of children and adolescents. Youth with chronic medical conditions are at an increased risk of psychological distress and reduced health-related quality of life, and report rates of mental illness up to double that of their physically healthy peers. Accessible, evidence-based interventions for young people with chronic illness are urgently required to improve their mental health and daily functioning. Self-compassion involves taking a mindful, accepting approach to difficult experiences, being aware that one is not alone in one’s suffering, and being kind and understanding with oneself during challenging times. Self-compassion shares strong associations with mental health outcomes among young people and preliminary work indicates that interventions that build self-compassion have the potential to substantially improve youth mental health. Self-compassion is also associated with better physical and mental health outcomes among individuals living with CMCs. While face-to-face self-compassion training is available, there are several barriers to access for youth with CMCs. Online self-compassion training potentially offers an accessible alternative for this high-risk group.

**Methods:**

Self-Compassion Online (SCO) is a self-compassion program that has been tested with a non-clinical adult group. For the proposed trial, a reference group of youth (16–25 years) with chronic illness reviewed the program and proposed adaptations to improve its suitability for youth with chronic illness. In alignment with the SPIRIT Checklist, this paper outlines the protocol for a CONSORT-compliant, single-blind randomised controlled trial to test the efficacy of the adapted program, relative to a waitlist control, for improving self-compassion, wellbeing, distress, emotion regulation, coping and quality of life among young Australians with CMCs. Mechanisms of action and feasibility of SCO will be analysed using quantitative data and participant interviews, respectively. Finally, cost-utility will be analysed using health-related quality of life data.

**Discussion:**

The SCO program could provide a scalable solution for improving psychological outcomes and quality of life among youth with chronic illness. The proposed trial will be the first to determine its efficacy for improving these outcomes, relative to waitlist control.

**Trial registration:**

The trial was registered on the Australian New Zealand Clinical Trials Registry on the 11th April 2019, ACTRN12619000572167.

**Protocol version:** Version 2, 21 December 2019.

## Background

Chronic medical conditions (CMCs) are those that last longer than 6 months and are likely to require medical follow up for more than a year [47]. Included under this umbrella are conditions such as diabetes, asthma, arthritis, and cancer. Prevalence of CMCs among children and adolescents is increasing and, depending on which conditions are included, prevalence estimates of CMCs in children and adolescents ranges from approximately 10–30% [25, 63, 65]. Estimates from population-based studies in the United States have found that over 30% of adolescents report one or more chronic conditions [46, 59]. In addition to placing substantial burden on the healthcare system, CMCs can carry substantial impacts for affected young people and their families. For example, young people with CMCs face many significant impacts on daily functioning, including pain, fatigue, and problems with sleeping patterns, social functioning, family relationships, school and occupational performance [53].

Youth with CMCs are also at substantially increased risk of mental illness [16], with population-based studies indicating that children with at least one chronic physical condition are 62% more likely to have a mental illness than those without chronic physical conditions [58]. A recent study found that 35.3% of young people in the general population have experienced co-occurring mental and physical disorders [59]. Findings from studies with adults also consistently demonstrate the co-occurrence of mental and physical disorders [15] and highlight the impact of such comorbidities on quality of life [5], disease management, and cost of care [66]. Such findings demonstrate the need to support psychological wellbeing among individuals with CMCs, particularly during adolescence and young adulthood, when individuals undergo multiple transitions [3] and experience heightened vulnerability to psychological distress [54].

Systematic reviews of psychological interventions for adolescents and young adults living with CMCs repeatedly highlight the need for accessible and age-appropriate interventions to address distress and promote wellbeing [51, 52]. Low uptake of existing interventions suggests the need for engaging alternatives that are theoretically sound. Self-compassion-based interventions are one such alternative. Self-compassion is associated with a range of advantageous mental health outcomes among young people [42], and emerging evidence indicates that interventions that build self-compassion have the potential to substantially improve youth mental health [8, 9]. Furthermore, several studies have shown an associated between self-compassion and better outcomes in adults with CMCs [26, 55, 56]. The current paper describes the protocol for a randomised controlled trial of online self-compassion training to promote wellbeing and reduce distress among young Australians with CMCs.

### Intervention approach and theoretical basis

The self-compassion intervention is based on the self-regulation model of chronic illness proposed by Lansing and Berg [38], which highlights the role of cognitive, affective and behavioural regulation in supporting resilience in the face of CMCs. Young people with CMCs are frequently required to deploy self-regulatory resources in order to maintain goal-directed behaviour, such as adhering to treatment regimens in the face of challenges or setbacks (for example, pain, fatigue, and social isolation [38];). Problematic emotion regulation and the use of maladaptive coping strategies are key drivers of maladjustment and negative psychological outcomes among patients with CMCs [13]. For example, emotion regulation strategies predict pain and functioning in youth with arthritis [14], and maladaptive coping strategies such as self-blame and rumination predict depressive symptoms in adolescents with diabetes [37]. As a result, interventions that promote self-regulation and teach adaptive coping skills have been identified as a priority for young people with CMCs [52].

Self-compassion is a parsimonious construct for supporting self-regulation across cognitive, emotional and behavioural domains, particularly in the context of chronic illness [55, 56] and psychiatric vulnerability [23]. Self-compassion is an adaptive form of relating to oneself in times of difficulty that involves three interconnected capacities: the ability to notice when one is struggling and to respond to difficult experiences in a balanced way; an appreciation of the “common humanity” inherent in challenges and awareness that one is not alone in one’s struggles; and the capacity to be kind and understanding with oneself during times of difficulty [45]. Self-compassion is a transdiagnostic resilience variable that promotes adaptive psychological and physiological self-regulatory responses to stress [2, 9, 20], and has been found to buffer the impact of stressful experiences on adolescent and young adult mental health, including traumatic events [68] and victimization [33, 34]. A recent meta-analysis found a large effect size for the inverse relationship between self-compassion and psychological distress across 19 studies of youth aged 10–19 [42].

Self-compassion is associated with less psychopathology and greater quality of life across multiple chronic illness groups, including among patients with cancer [49], HIV [10], epilepsy [12], inflammatory bowel disease (IBD), and arthritis [55, 56]. By promoting adaptive emotion regulation in the face of difficult experiences [23], self-compassion may support individuals to cope with the experience of chronic illness, as well as buffering the detrimental impact of self-stigma [29]. This has been demonstrated in a study of patients with IBD and arthritis, where self-compassion was found to predict adaptive coping, which in turn was associated with enhanced coping efficacy and reductions in perceived stress [55, 56]. Additionally, self-compassion may play a role in quality of life in individuals with CMCs as it is associated with increased engagement in health-promoting behaviours [57].

Previous studies of self-compassion training for adults with CMCs have demonstrated positive effects on mental and physical health outcomes. For example, a randomised controlled trial comparing self-compassion training with waitlist control for adults with diabetes found significant improvements in mental health and metabolic outcomes among the intervention group [26]. In addition, a randomised controlled trial of self-compassion training compared with an active control (relaxation) reported a 40% absolute risk reduction for health status in the intervention group for patients with fibromyalgia [43]. Further, previous work has found that self-compassion interventions improve mental health and wellbeing among healthy adolescents [8, 9, 28]. However, in each of these studies, the intervention has been delivered as a face-to-face, group-based intervention (approximately 2 h per week for 6–8 weeks). Arguably, this mode of delivery is not well-suited to many adolescents and young adults with CMCs, who may experience limitations in their capacity to access face-to-face services, as well as already bearing a high time and resource burden for medical treatment. Digital health interventions are an efficacious alternative to face-to-face care for youth mental health [48], although there is currently only low-quality evidence to support their use among young people with CMCs [60]. The current trial will examine a brief, online self-compassion training program to improve psychological wellbeing among young people with CMCs. Using a randomized design with a wait-list control, we will provide evidence regarding efficacy, acceptability, and cost-utility. A waitlist control was considered appropriate given that this and exploratory study and we are also interested in testing the feasibility and acceptability of the intervention with the target group. The waitlist control group will access treatment as usual.

Self-Compassion Online (SCO) is a self-guided web-based program grounded in an emotion regulation model of self-compassion [24]. The program draws on key elements of the mindfulness and acceptance-based model of therapeutic change, including psychoeducation, meditation, and self-reflective exercises [24]. The SCO program has been piloted with a non-clinical, primarily young adult sample, who reported significant pre-post improvements in depression, stress, emotion regulation, happiness, and self-compassion [24]. Feedback from the pilot of the program led to revisions in terms of length (from six to 4 weeks) and complexity of content. For the purposes of the current trial, the program was further adapted to make it appropriate for adolescents and to ensure relevance and application to the challenges of living with a CMC. The adapted program was reviewed by members of a Youth Reference Group (YRG) consisting of eight young people (16–25 years) living with a CMC. The YRG provided feedback about each of the four modules in the program, via a combination of online surveys and online consultations. Two online consultations were held; one in which initial feedback was sought, and a second, in which the adaptations made were discussed to ensure alignment with feedback. Based on this feedback, the SCO content and structure were further refined prior to the current trial. An overview of the revised intervention – Self-Compassion Online – Chronic Medical Conditions (SCO-CMC) is shown in Table 1.

**Table 1:** Self-compassion Online Program Overview

Module	Content
Befriending Yourself	Introduction to self-compassion Understanding the inner critic Understanding relationships between thoughts, feelings, and behaviours Being a friend to yourself
Calming Your Mind	Three affect regulation systems Anchoring your mind Training your attention (breath-focused meditation) Alternative anchors (five senses practice) Coping with difficult feelings
Understanding your Strengths	Understanding character strengths Using your strengths Self-acceptance Connecting with others Loving Kindness Practice
Cultivating a Meaningful Life	Appreciating the good Identifying your values Using your values Values and self-compassion Moving forward

### Aims and hypotheses

The primary aim of this trial is to determine whether SCO-CMC can produce significant improvements in self-compassion, emotion regulation and coping, relative to a waitlist control, among youth with CMCs (16–25 years). In addition, we will investigate whether the intervention is associated with improvements in psychological distress, wellbeing, and quality of life and whether improvements in self-compassion, emotion regulation and coping mediate the effect of the intervention on wellbeing, distress and quality of life. A secondary aim of the study is to determine the cost-utility of the program by calculating the incremental cost-utility ratio for the intervention compared to usual care. Finally, we also aim to examine feasibility of the program, by examining enrolment and retention rates, and determine satisfaction and acceptability of the program by interviewing young people who took part.

It is hypothesized that relative to waitlist control, the SCO-CMC group will:

1. Report significant increases in self-compassion and approach coping, and significant decreases in emotion regulation difficulties and avoidant coping
2. Report significant reductions in symptoms of psychological distress
3. Report significant improvements in quality of life and wellbeing

It is also expected that improvements in self-compassion, emotion regulation, and coping will mediate the relationship between treatment group and reduced psychological distress, as well as improved wellbeing and QoL.

## Methods/design

### Ethics approval

The study protocol was approved by the Human Research Ethics Committee at Curtin University HRE2019–0386.

### Trial design and study setting

The proposed study is an exploratory single-blind CONSORT-compliant randomised controlled trial, comparing online self-compassion training (SCO-CMC) with a waitlist control group (WLC). The intervention and data collection will be online, with data collected from Australian residents only.

### Participants

Participants will be *N* = 96 Australian adolescents and young adults (48 per group), aged between 16 and 25 years, who self-report a current diagnosis of a CMC (for example arthritis, asthma, cancer, cystic fibrosis, diabetes, epilepsy, haemophilia, IBD, lupus, multiple sclerosis, and myalgic encephalomyelitis). Additional inclusion criteria are as follows (1) Australian resident; (2) fluent English speaker; (3) regular access to the Internet; (4) able to access online video and audio content.

### Sample size

Using medium effect sizes, 40 participants per group are required for power = .80 and α = .05 [21]. To allow for typical treatment drop out of approximately ~ 15–20% [17] a target of 48 people per group has been set. This sample size is also sufficient to detect a medium effect for the purposes of the mediation analysis [27].

### Outcome measures

#### Psychological distress

Psychological distress over the past 30 days will be measured using the Kessler 10-item psychological distress scale (K-10 [36];). The K10 is a self-report measure comprising 10 items on a 5-point scale. Total scores range from 10 to 50; scores above 15 indicate moderate to severe psychological distress, while scores greater than 20 indicate higher likelihood of mental disorder [1].

#### Self-compassion

Self-compassion will be measured using the Short Form of the Self-Compassion Scale (SCS-SF [50];). This self-report scale consists of 12 items designed to measure the six subcomponents of self-compassion: mindfulness, overidentification, common humanity, isolation, self-kindness (e.g. “I try to be understanding and patient towards those aspects of my personality I don’t like”) and self-judgement; each item is rated on a 5-point scale. Item scores are used to generate a total self-compassion score, which has a high correlation with the total score of the long form Self-Compassion Scale [50]. Psychometric studies have demonstrated the reliability and validity of both forms of the Self-Compassion Scale with adolescents and young adults.

#### Emotion regulation difficulties

Emotion regulation difficulties will be measured using the Difficulties in Emotion Regulation Short Form (DERS-SF), a widely-used 18-item measure of emotion regulation problems [35]. This self-report scale measures difficulties with emotion regulation along six dimensions: (1) awareness of emotions; (2) emotional clarity; (3) acceptance of emotions; (4) access to emotion regulation strategies; (5) ability to engage in goal-directed behaviour when experiencing negative emotions; and (6) impulse control in the face of difficult emotions. The DERS-SF provides a total scale score for emotion regulation difficulties and is a valid and reliable measure of difficulties with emotion regulation in both adolescents and adults [35].

#### Coping

Coping will be measured using the brief COPE [11], a 28-item self-report measure of coping strategies which is a short version of the original COPE scale. Respondents rate items (e.g. “I take action to try to make the situation better”) across 14 subscales, using a 4-point scale. The subscales can be categorised into those representing approach coping (active coping, emotional support, use of informational support, positive reframing, planning, and acceptance) and those representing maladaptive coping (denial, self-distraction, substance use, venting, self-blame, and behavioural disengagement) subscales. Avoidant coping has been associated with poor physical health outcomes in adults with chronic medical conditions [18]. The Brief COPE has good internal reliability [11] including among adolescent samples [30].

#### Wellbeing

Wellbeing will be measured using the World Health Organization Wellbeing Index (WHO-5), a widely-used, validated measure of subjective wellbeing designed for use in clinical trials [62]. The WHO-5 measures positive psychological well-across 5-items using a 6-point scale, and scores are used to obtain a percentage score ranging from 0 to 100. The WHO-5 displays acceptable psychometric properties among adolescents [7] and adults [6].

#### Quality of life

The Assessment of Quality of Life – 6 Dimension (AQoL-6D) is a generic, multi-attribute utility, preference-based measure that will be used to assess health-related quality of life [44]. It is an adaptation of the adult AQOL-6D utility instrument and has 20 items across six dimensions - independent living, mental health, coping, relationships, pain, and senses. There are scoring algorithms for both Australian adults and adolescents for the AQoL-6D [44].

#### Health resource use

A health resource use questionnaire was developed for the purposes of the study, based on the core items for a standardized resource use measure highlighted by Thorn et al. [61]. This measure asks about frequency of health service use, frequency and length of stay for hospital admissions, and frequency and dose of medication use over the past month. It will be used to collect resource use data for the purposes of the cost-utility analysis (CUA).

#### Program engagement and adherence

Program engagement will be measured using online program metrics, including log-ins, time spent in program, and module completion, as well as self-report of engagement with practice outside of program. These variables will be treated as a measure of program feasibility and will also be explored as potential moderators of treatment effects. Program referrals and attrition data will also be gathered as a measure of program feasibility.

### Procedure

#### Recruitment, randomisation and allocation

**Fig. 1:**
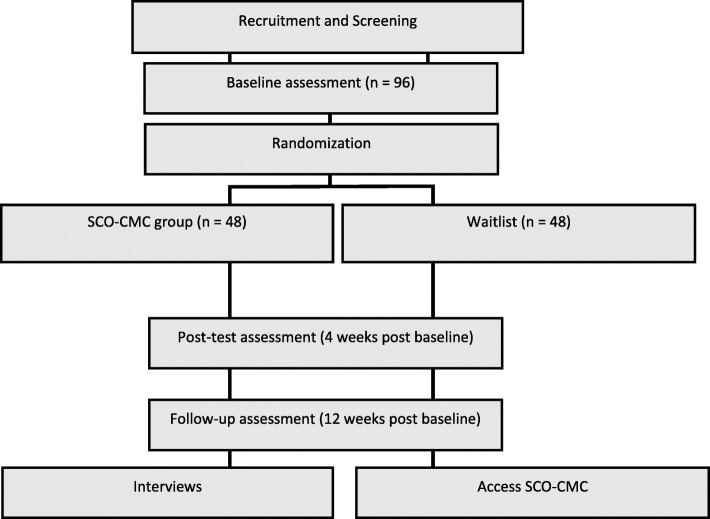
An overview of the Study Design

A diagram of the study design is shown in Fig. 1. Participants will be recruited from medical services and chronic illness networks; social media and online communities across Australia. Interested participants will be directed to a website which will outline study eligibility and will contain a link to the online consent form and screening measures. Participants who meet inclusion criteria will be randomly assigned a participant code, in order to link data across time points while preserving anonymity. They will proceed to an online pre-test questionnaire which will gather demographic and health resource use data (re-administered at post-test and follow-up), as well as the outcome measures. To promote retention participants will be informed that they will receive AUD$20 after completing the post-test surveys, and an additional $20 after they have completed the follow-up surveys. Participants will also be able to opt-in to receive a copy of the study results via email.

Following completion of the pre-test, participants will be randomised to conditions using the Qualtrics randomiser function. Participants in both arms of the trial will be contacted by a research assistant shortly after completing the baseline survey, who will inform them of their condition allocation, reiterate study procedures and answer any questions: as participants are recruited on a rolling basis, those assigned to SCO-CMC will receive access to the program as soon as the research assistant has made contact with them. At four and 12 weeks after completing baseline measures, all participants will be sent an email asking them to complete the outcome measure battery again. Following completion of the third set of measures, participants in the WLC will be contacted by the research assistant, who will provide a code for program access. All participants will receive up to two reminders to complete the measure battery at each time point; participants enrolled in the intervention will also receive an email or text reminder 2 weeks post enrolment to encourage them to continue progressing through the program.

#### Qualitative data collection and analysis

At baseline, participants will be randomly selected to be invited to participate in a telephone interview to discuss their experience of participating in the SCO program: participants will be invited to participate in the interview following the third assessment point. Forty participants will be randomly selected at baseline and invitations will be issued until ten interviews are complete. Baseline selection is designed to ensure that interviewed participants include those who may have disengaged from the program. Interviews will be conducted by the research assistant and will explore the impact of the SCO-CMC program on the lived experience of young people with CMCs, identify potential mechanisms of change not assessed in quantitative measures, and gather data on potential iatrogenic effects. Interviews will also be used to gather detailed information on program satisfaction and feedback regarding potential revisions. Finally, qualitative data will also be collected by the online program (i.e. in the interactive exercises which use digital forms for participants to complete exercises). We will conduct thematic analysis of this data to identify key themes regarding program benefits, challenges, and application of program strategies to participants’ lived experience.

#### Data monitoring, management, and post-trial care

No data monitoring committee will be engaged given that the intervention is low risk and participants are able to access care as usual while participating. Interim analyses will not be conducted. Study investigators will permit trial-related audits in line with Curtin University Human Research Ethics Committee requirements and approvals. Protocol amendments will be submitted to the Curtin University Human Research Ethics Committee, and, if approved, communicated to participants via email.

A research assistant will be engaged to ensure blinding of the data analyst to group allocation, and data will be managed in line with Curtin University data management guidelines. Participant email addresses will not be stored with any of the trial data. Participants will be encouraged to liaise with the research assistant if they wish to withdraw from the trial or if they wish to be directed to additional psychological support at any point. Information about free online and telephone-assisted psychological support is also provided to participants on the participant information sheet, and within the SCO-CMC program.

### Statistical analysis

#### Tests of intervention effects

We will perform Intention to Treat and Per Protocol analyses, conduct a sensitivity analysis [39], and compare complete cases versus cases lost to follow-up on baseline characteristics and scores on clinical measures, by randomisation group [64]. GLMM Mixed Model Repeated Measures will compare change in outcome measures across conditions, and time points, while controlling for random effects including age and gender. The group*time interaction will be calculated as a test of intervention effects. Using DASS-21 scores participants will be classified into outcome categories (Recovered, Improved, Unchanged, Deteriorated) according to Reliable Change Index and Clinical Significance of change at post-treatment and follow-up [32], to determine whether the SCO-CMC and control groups differ in clinically significant treatment outcome over time (Cochrane’s Q).

#### Mediating mechanisms

Mechanisms of action will be tested using multiple mediational analysis with bias-corrected bootstrap samples in MPlus, to test total and specific indirect effects. This is the most powerful test of mediation, requiring a sample of 71 participants to detect a medium effect [27].

#### Cost-utility analysis

CUA calculates the ratio between the costs of intervention and gains – in terms of quality-adjusted life years – against the costs and gains associated with a comparator. An incremental cost-utility ratio is calculated by dividing the incremental costs by the incremental effects using the following formula:

(mean Costs _intervention_ − mean Costs _comparator_)/(mean QALYs _intervention_ − mean QALYs _comparator_).

We will calculate the cost-utility of SCO-CMC compared with treatment as usual, from the perspective of the Australian health system. This assumes that the health system pays for the cost of the intervention and includes other costs covered by the health system only. We will calculate the cost of SCO-CMC using a bottom up approach. This will include the time taken for an assistant to enrol and contact participants as well as the cost of hosting the online program. Costs between baseline and follow-up will be calculated for each participant by summing costs measured using the resource use questionnaire and valued using Medical Benefits Schedule data (for visits to healthcare professionals), National Hospital Cost Data Collection data (for hospitalisations) and Pharmaceutical Benefits Schedule data (for pharmaceuticals). For the SCO-CMC group, total costs will also include the cost of the intervention. The number of QALYs per patient will be calculated by multiplying the AQoL-6D utility score by the appropriate time period. A base-case intention-to-treat analysis will be performed using all participants. To evaluate the impact of uncertainty on the cost and QALY estimates for each group, we will use bootstrapping with 5000 replications. These findings will be plotted on a cost-utility plane, which comprises four quadrants. We will also plot a cost-utility acceptability curve. This shows the probability that the intervention is SCO-CMC is cost-effective when compared with treatment as usual at different willingness-to-pay thresholds.

## Discussion

This paper describes the protocol for a randomised controlled trial designed to assess the efficacy of an online self-compassion training program for promoting self-compassion and improving emotion regulation, coping, psychological distress, wellbeing and quality of life in young people (16–25 years) living with a CMC. The intervention approach is based on a self-regulation model of CMCs, which highlights the importance of cognitive, emotional and behavioural self-regulation for supporting self-management and optimising physical and mental wellbeing [38]. The four-week SCO-CMC program is a modified version of the online self-compassion training program described in Finlay-Jones et al. [24], and was adapted following review and consultation with a YRG comprising of eight 16–25 year olds living with at least one chronic condition.

Online self-compassion training holds promise as a highly-accessible, brief intervention to enhance mental health among this group. Self-compassion is a robust transdiagnostic predictor of a range of adaptive outcomes [40, 42], and self-compassion training is associated with improved physical and mental health among adults with chronic illness [26]. Online training is a flexible, low-cost, sustainable mode of delivery that is appropriate for young people with chronic illness, whose capacity to access face-to-face services may be limited. Previous research supports the notion that psychological outcomes among people living with chronic conditions can be improved following web-based intervention, although the majority of research has been conducted with adults [19].

While there is preliminary evidence to support the efficacy of self-compassion-based interventions online [24, 41], this will be the first study to trial a self-guided online self-compassion intervention with chronically ill adolescents and young adults. In addition to gathering preliminary data on the efficacy of this program for improving self-compassion and wellbeing-related outcomes among the target group, the proposed study will also gather valuable data on program engagement and experiences in the program among the target group. This is important given that a recent systematic review noted a lack of data regarding the feasibility, acceptability, and efficacy of self-compassion interventions for younger populations [22]. Additionally, previous studies of online mindfulness-based interventions have highlighted the need for a better understanding of *how* people engage in such interventions [4]. Conducting qualitative analysis of responses to online exercises and conducting follow-up interviews with participants (including, potentially, those who drop out of the program) will allow us to gain insight into how young people with chronic conditions make sense of self-compassion training and apply it to their lives.

This study also has potential to extend our current theoretical understanding of the mechanisms of action underlying self-compassion-based interventions. Analysis of mediating mechanisms in self-compassion intervention studies is extremely limited [22], although cross-sectional studies have highlighted the role of emotion regulation as a mediating mechanism in the relationship between self-compassion and a range of mental health outcomes [23, 31]. Further, previous work indicates that self-compassion improves emotion regulation and adaptive coping among individuals living with chronic illness [55, 56, 67]. This study will extend this work by examining whether self-compassion training promotes self-compassion and improved emotion regulation and coping, and whether these changes lead to better psychological outcomes in the target group.

Finally, establishing efficacy of the SCO-CMC program for young people with chronic illness could provide a scalable solution for improving mental health and quality of life among this sizeable population. The proposed study will integrate cost-utility analysis to determine the incremental cost-utility of the program compared to treatment-as-usual. This will provide a preliminary understanding of whether the program provides value-for-money compared with usual care, as well as piloting methods for assessment of resource use in a heterogenous adolescent and young adult chronic conditions population.

## Data Availability

No datasets were analysed during the preparation of this protocol paper. Materials used are available, upon reasonable request, via contact with the corresponding author. Ethics approval has been granted for deidentified data from the trial to be deposited in an open access data repository (https://osf.io/2hak4/?view_only=60888a7e6f814ebaac719c873615a3f3). All investigators will have access to the deidentified dataset, however only the research assistant will have access to identifying information. Results of the study will be submitted for publication following study completion.

## Abbreviations

CMC: Chronic Medical Conditions
IBD: Inflammatory bowel disease
QALYs: Quality-adjusted life years
SCO-CMC: Self-Compassion Online – Chronic Medical Conditions
